# Privacy Issues in the Development of a Virtual Mental Health Clinic for University Students: A Qualitative Study

**DOI:** 10.2196/mental.4294

**Published:** 2015-03-31

**Authors:** Amelia Gulliver, Kylie Bennett, Anthony Bennett, Louise M Farrer, Julia Reynolds, Kathleen M Griffiths

**Affiliations:** ^1^ National Institute for Mental Health Research Research School of Population Health The Australian National University Canberra Australia; ^2^ Young and Well Cooperative Research Centre Melbourne Australia

**Keywords:** university, student, mental health, internet, virtual clinic, qualitative

## Abstract

**Background:**

There is a growing need to develop online services for university students with the capacity to complement existing services and efficiently address student mental health problems. Previous research examining the development and acceptability of online interventions has revealed that issues such as privacy critically impact user willingness to engage with these services.

**Objective:**

To explore university student perspectives on privacy issues related to using an online mental health service within the context of the development of an online, university-based virtual mental health clinic.

**Methods:**

There were two stages of data collection. The first stage consisted of four 1.5-hour focus groups conducted with university students (n=19; 10 female, 9 male, mean age = 21.6 years) to determine their ideas about the virtual clinic including privacy issues. The second stage comprised three 1-hour prototype testing sessions conducted with university students (n=6; 3 male, 3 female, mean age = 21.2 years) using participatory design methods to develop and refine a service model for the virtual clinic and determine student views on privacy within this context.

**Results:**

The students raised a number of issues related to privacy in relation to the development of the university virtual clinic. Major topics included the types of personal information they would be willing to provide (minimal information and optional mental health data), concern about potential access to their personal data by the university, the perceived stigma associated with registering for the service, and privacy and anonymity concerns related to online forums contained within the virtual clinic.

**Conclusions:**

Students would be more comfortable providing personal information and engaging with the virtual clinic if they trust the privacy and security of the service. Implications of this study include building the clinic in a flexible way to accommodate user preferences.

## Introduction

Mental disorders are at their peak in young people aged 16 to 25 years [1], a time when many young people are enrolled in university [2]. University life can expose students to additional stressors [3], which may impact negatively on their mental health. Mental disorders are responsible for a significant disability burden in university students [4], with a recent US study reporting a 12-month mental disorder prevalence level of almost 50% in university students [5]. Mental disorders can have severe consequences if untreated, including disability [6,7], suicide [8], lowered quality of life [9], and, for students, a greater risk of dropout from their education [10]. Despite this, less than one-fifth of students with mental health problems use mental health services [5].

The tertiary education setting is a unique environment in which to provide both large-scale preventive and treatment interventions for mental health problems [11]. Mental health services in a university setting tend to be delivered face-to-face [12], which may limit their cost-effectiveness and scalability. In addition, the high clinical load often experienced by staff at university health clinics and economic burden on the university [13] restrict the number of students who can access face-to-face care. Technology-based interventions show promise for treating common mental disorders such as depression and anxiety in university students [14]. Interventions delivered via the internet can be continuously available, accessed anonymously, cost-effective [15,16], scalable, and broadly disseminated [17,18]. University students use the internet frequently and are highly likely to search for information about mental health online [19]. Moreover, Internet interventions may be viewed as less stigmatizing than traditional approaches to care [20,21], with university students being concerned with perceived stigma associated with accessing on-campus counseling services [22]. Therefore, there is a potential role for a university-based online service that has the capacity to complement traditional services to more effectively and efficiently address student mental health problems.

A key issue in the development of an online health service (and indeed, any health service) is the user’s experience of privacy [23,24]. Research outside the university setting has investigated privacy issues relating to the security of user personal information [25], predominantly in the context of consumer portals in medical settings [26-31]. One study examined consumer attitudes towards privacy when provided with online access to their medical records [29]; another explored concerns about privacy in the development of personal health records for veterans [30]. All studies identified privacy as a significant concern for users of these services. Although these studies have focused on adults in the community, it is possible that privacy is an even greater concern for students, particularly those studying certain disciplines like law or medicine [32,33]. For example, students may fear being labeled with a mental health condition or being discontinued from their course by the university [34]. Little qualitative research has been conducted to investigate privacy issues among university students or the types of personal information that students are comfortable providing with respect to their mental health. This study seeks to address the gap in knowledge.

## Methods

### Overview

This study was conducted within a larger project involving the development of a university-specific virtual mental health clinic designed to provide support to students across the mental health intervention spectrum (from awareness and prevention to treatment and relapse prevention). Participatory design methods attempt to involve all stakeholders (eg, end-users, employees, administrators) in coproducing a service [35]. In this project to date, these methods have included focus groups and prototype sessions which were used to engage students (end-users) in the development of the virtual clinic. Using participatory design methods may increase uptake of the service among students and foster a sense of empowerment and ownership of the service [36].

This research is taken from the first two stages of the project. The first stage comprised qualitative focus groups, which broadly examined the topic of mental health help-seeking online, and the second stage consisted of iterative prototyping of model versions of the clinic and feedback cycles conducted with students. The focus groups and prototype testing sessions were structured and based on predetermined questions to aid the development of the virtual clinic while allowing for other topics to be discussed as they arose. A list of questions for the focus groups and prototype sessions is provided in Multimedia Appendix 1.

### Study Sample

The overall sample for focus groups and prototype sessions consisted of 25 students (13 female, 52%) from The Australian National University (ANU). The mean age was 21.5 years (range 18-24).

#### Focus Group Sessions

This project stage involved conducting four focus groups with 19 university students (10 females, 9 males; n=5, 5, 5, 4) to determine their views on online help-seeking for mental health problems and ideas about components of the virtual clinic and how it might function. The mean age of the focus group sample was 21.6 years (range 19-24). Each group lasted approximately 1.5 hours. Detail on the methods for the focus groups have been published previously [36].

#### Prototype Sessions

This stage of the research involved conducting three 1-hour prototype testing sessions with 6 university students (3 females, 3 males; *n*=1, 2, 3) to engage students in the development and refinement of a service model for the virtual clinic. During these sessions, participants provided feedback on a prototype version of the virtual clinic, developed by the research team based on the previously conducted focus groups; relevant literature; and clinical, empirical, and information technology best practices. The prototype presented to the participants depicted a student, how the student may encounter and use the clinic, as well as example features of the clinic and how the clinic may interact with service providers (eg, a university counseling center). This process was iterative and each session contained a new prototype for testing, which took into account input from participants in previous sessions and further elements to be tested as determined by the researchers. The mean age of the prototypes sample was 21.2 years (range 18-23).

All groups were facilitated by LF with note-taking by either AG or JC and were conducted onsite at the National Institute for Mental Health Research at ANU with the exception of one focus group which was held in a meeting room at a student hall of residence. The researchers mutually agreed that data saturation (no new themes emerging) had been reached after four focus groups and three prototype sessions. Students were recruited for both stages via email invitations sent to a list of students who provided their name and email address at previously held mental health awareness events. In addition, a snowball sample was implemented whereby potential participants were encouraged to invite other students to participate. Finally, the researchers also contacted a senior resident at a student residential hall for assistance in advertising the focus group to other residents.

### Ethics

All participants provided written consent after reading an information sheet. A cinema pass was supplied to students on completion of the focus group in appreciation for their time. Ethical approval for both stages of the research was granted by the ANU Human Research Ethics Committee (focus groups, 2012/520; prototype sessions, 2013/491). Ethical issues that were addressed in these submissions included protecting participant privacy by instructing participants to maintain confidentiality of the content discussed, ensuring any distress that occurred during groups was appropriately managed by a trained psychologist, and protecting individually identifiable data. No problems related to these issues arose. All focus groups and prototype sessions were audiotaped and transcribed verbatim.

### Analyses

Two researchers (AG and JG) identified and coded quotes that were relevant to the topic of privacy. Subsequently, thematic analysis [37] was broadly used by the first author (AG) to classify participant statements and ideas into themes using grounded codes [38]. The number of comments and ideas in the discussion created on each topic during the focus groups and prototype sessions was used to determine the major themes and subthemes in the data. In the findings below, the quotes that best represented each theme are reported and the participants are identified by gender, participant number, and group type (ie, *F1_FG* = female participant number 1, focus groups; M2_P = male participant number 2, prototype sessions).

## Results

### Student Perspectives

**Figure 1:**
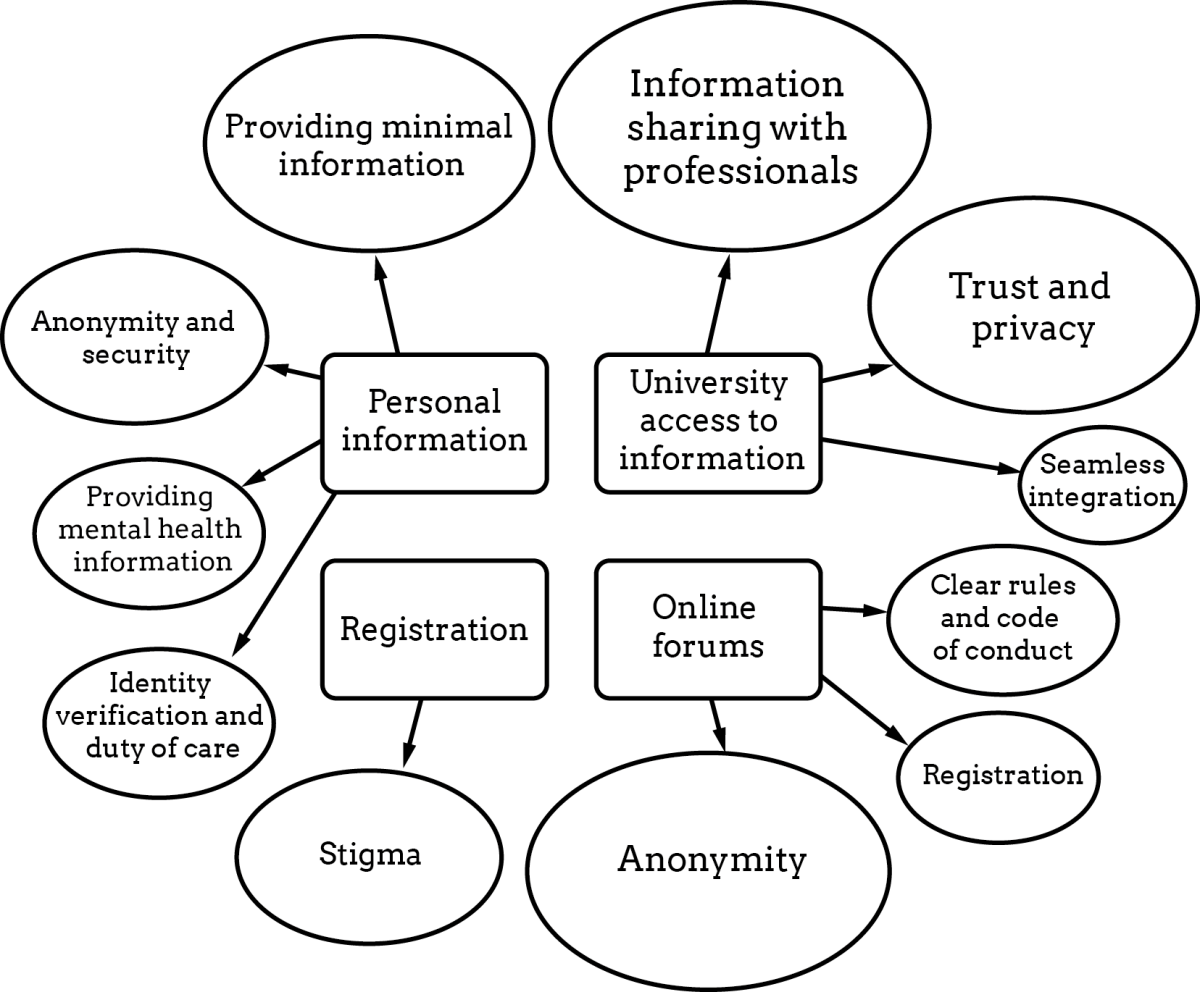
Privacy themes raised in relation to the virtual clinic by students. Larger shape indicates a greater volume of discussion generated in the topic.

Figure 1 presents a concept map of the privacy themes raised in relation to the virtual clinic, including (1) what personal information participants would be willing to provide, (2) concern about the university’s access to information or personal data, (3) stigma associated with registration, and (4) privacy issues related to online forums within the virtual clinic. The results below present the data according to major and minor themes. Each theme is ordered by its relative importance as judged by the number of comments and ideas generated for each topic across the focus group and prototype session discussions.

### Personal Information

#### Providing Minimal Information

Participants considered the types of personal information required to create an account to be very important. They believed that providing minimal demographic information for a service such as a virtual clinic would be acceptable (eg, age range, gender, international/domestic student status, study load status, on- or off-campus residence). Overall, participants felt it was important that the information was relevant to the service and, most importantly, that it was collected for a reason: “Things that may not be relevant shouldn’t be asked.” [M1_FG] Because of this, they found it acceptable for the virtual clinic to ask for information that would enable content to be more tailored to their experience and emphasized that the site should justify to the user why the information is needed,

[Explaining] why we’re giving that information, so if it is like those type of things that can help…target, like, the site or something to you, and instead of registering your account it’s just…help us target it for you.

F1_P

However, this did not extend to any personally identifying information such as age or birth date: “I just don’t think people would want to come to it with a birth date and, identifying themselves.” [F1_P] Additionally, some participants were happy to provide area of study, whereas others felt that would be too identifying. Overall, they felt it was extremely important that all information provided should not be compulsory: “What personal information you should be able to be comfortable providing…most of the things should be optional.” [M2_FG]

#### Anonymity and Security

In general, the participants believed that they would be more likely to provide information anonymously: “The more anonymous it is, I think, the more information people are willing to give.” [M3_FG]

Participants also indicated that they would be more likely to share information if they trusted the security of the website,

I mean it depends on who gets to see what and on whether I trust the security of the site or not. Um, because, I’d sort of be quite happy to put, you know, most personal information, like client details and personal information…if I, you know, trusted that control to work.

M4_FG

I’m actually more than willing to put in every single thing as long as I know that the whole system is safe.

M1_FG

In general, even though the participants knew confidentiality and security measures were taken, they still had concerns that generally, “the Internet just seems kind of insecure.” [F2_FG]

#### Identity Verification and Duty of Care

Participants indicated that it was important that the system could verify that a user was entitled to register for the virtual clinic (ie, being a student) to ensure user safety: “So you’re not talking to some creepy person.” [F3_FG)] They also thought that it was important that users could be contacted (via email) by the providers of the virtual clinic site. Thus, they considered that being able to be identified in some way was necessary: “I guess other, really other than name…pretty much a valid email… you shouldn’t have to provide much more information than that.” [M2_FG] Additionally, they recognized that duty of care issues for the providers of the clinic were important and that users may need to be contacted in emergency situations,

If they’re in crisis, maybe…with their permission passing on a phone number or something to the CAT [Crisis and Assessment] team and getting them to give them a call.

F3_FG

However, they acknowledged that if users did not want to provide information then perhaps “the best you can do is say like…‘we’d love it if you called this CAT team number’…they never have to give away their information online.” [M5_FG]

#### Providing Mental Health Information

Participants felt that quizzes about mental health were acceptable. However, again they made it clear that such quizzes should be optional,

I think if you could have like a pop-up quiz…‘we’d like to invite you to fill in this short quiz–it’s entirely up to you whether to fill it in or not,’ because I think that matter of choice is important. Don’t shove it down their throat.

M6_FG

### University and Other Access to Personal Information

#### Information Sharing With Professionals

The participants felt that it was important to accommodate personal preference for information sharing between organizations. For example, some students would want mental health professionals to have access to their account: “If you could connect with a specific psychologist using this site and then they can actually review your material directly through this space. That could be useful.” [M3_FG] Others felt this access for their psychologist might save time in their counseling sessions: “The counseling center makes you go on a computer anyway…and fill out some kind of form…it could just do this as well.” [M7_P]

Although participants were generally supportive of information sharing with professionals, they were concerned about the security of this data,

If you have information on there that has been looked at in one way by professionals or something, you probably don’t want that information being put out there for all the other users on the site to see.

F4_FG

The participants agreed that no information should be available without a person’s consent and that taking into account personal preference was important: “If the user…intends to keep their information private, private or confidential from those professionals…it would be important if you consider the concerns of the user first before you offer any kind of solution or suggestion.” [F5_FG]

#### Trust and Privacy

The participants raised several concerns about other university staff being able to access their personal information. They felt that a perceived lack of privacy had the potential to deter students from using the virtual clinic: “Fears about the lack of privacy is going to be a huge issue.” [M3_FG] Because of this, they felt it was extremely important that the virtual clinic website was trustworthy and that any information shared was kept confidential: “Knowing that your information isn’t going to feedback elsewhere—whether it’s to like academics or family…knowing that the information you shared is secure.” [F6_FG] They also believed that the virtual clinic needed to be transparent in communicating where this information could potentially be shared: “I guess it depends. I think just communicating…where it’s going to go is really important.” [F6_FG] It was also considered important that the list of terms and conditions that explained where their information would go was brief and easy to understand: “Short and succinct… not too lengthy, ‘cause as soon as it becomes more than like a paragraph, no one reads it.” [F2_FG]

An idea that arose repeatedly across groups was that students would want to the ability to set and maintain their own privacy settings for different groups (eg, clinicians, friends, other users),

Different levels of accessibility to information. You can set what information you show to certain people. So you can make it entirely public, you share all your information with anybody and everybody or you can restrict access so only certain people can see.

M6_FG

In relation to this idea, the concept of requiring a password to gain additional access to their virtual clinic accounts also emerged: “You could customise different levels. You know, when do I need a password?” [M7_P]

#### Seamless Integration

The participants suggested that registering their own account for the virtual clinic (with an anonymous username and password) would minimize the need for their account to be linked to any identifying details. Conversely, some participants considered that creating a new account was onerous and would prefer it to be generated using their existing log-in information for their online student account. They also noted that there could be seamless log-in across all university services as they found it “irritating when you have to swap from [one online university service to another]” [F7_FG] and that they were already exposed to “a lot of integrated stuff” [M7_P] (eg, student email, other online student services). However, participants noted that they often allowed their peers to log in to their online student accounts to access printing and other services, so they felt this could compromise the privacy of their personal information contained within the virtual clinic: “My friends and I, like, when you run out of print quota…you give someone else your login so…if they had a different password for this thing, I would feel more comfortable.” [F8_P] The idea of a guest account or non–signed-in access was suggested as a potential way of allowing students to access material with which they may not otherwise engage,

I think it’s important to have your username account but also a guest account so if you don’t want to do something which someone else can see, you can use the private guest account, which won’t record your information.

M8_FG

### Stigma of Registration

The participants felt that actively registering for the service could increase their sensitivity to stigma as it indicated that they identify as having a mental health problem,

I think it’s the fear of like, again like people have that stigma attached, like if you register people are going to like, somehow you’re going to be…identified as a person who has a problem...it’s like saying ‘I need help.’

M10_P

The participants felt that one way of overcoming this was to seamlessly integrate the virtual clinic with other university services, as mentioned above. This would involve all students having an automatically registered account with the virtual clinic, alongside other freely available online student services that students already access (eg, course administration interfaces) so that “every student can have an account” [F9_P] and “you don’t have to use them, no one’s saying you have to, it just means every student is on the system.” [F1_P] Participants suggested that they could create an account name so that any activity on the virtual clinic would not be identifiable by other users of the website: “you log in with your uni ID but when you make an account, it will make sure you make an anonymous profile name.” [M1_FG]

The participants indicated that being able to hide their visibility on the site was paramount: “You don’t want other people to see, ‘Oh this person is online—she must be going through something.’” [M8_FG] Because of this, some participants felt that parts of the virtual clinic should be visible at all times (eg, general information), whereas other sections should be restricted to registered users (eg, people’s profiles). Other participants thought that all virtual clinic content should be available without having to register: “I’d want to have the option of being able to explore it without signing up.” [F9_P]

### Online Forums

#### Anonymity

The participants thought that a forum would be an important element component of a virtual clinic for university students. Some students, as with registration, felt that it was important to have an anonymous username in forums: “To post on forums you need some kind of a username. If that could not be attached to your student number, then that would create that kind of sense of privacy.” [F1_P] However, other participants felt that certain students would prefer to not be anonymous: “Some people are quite comfortable talking about mental health issues and they don’t mind putting their name out there.” [F6_FG] Some participants felt that it was important for the system to be capable of doing both,

I guess that’s when you need the option to use it as anonymously as a tool for yourself, for your personal needs or as a networking device like I think it needs to do both.

M3_FG

The ability to disclose your identity to specific people was suggested by participants (“Do you want to share your details with this person?” [M10_P]), in order to enable users to meet up in person with larger groups of other students: “We can get together and talk about that, like over coffee, and then people can, yeah, disclose who they are online if they want.” [F1_P] However, participants acknowledged that there was a difficult balance between ensuring the privacy and security of personal information and providing an avenue for social interaction outside the clinic.

#### Clear Rules and Code of Conduct

The participants felt it was very important that the rules of the forum were clearly stated, including basic etiquette and what would happen if members posted any content about harming themselves or others,

Perhaps including something about that in the code of conduct like ‘we will contact you if you xyz.’

M3_FG

Cause you don’t want it to be out of surprise but if I know you’ll be contacting me then [that’s ok].

M8_FG

Some participants also liked the idea of private instant messaging between members within the forums but recognized that this could also be potentially unsafe: “Cause that would be the easiest way to bully someone—message them privately.” [F2_FG]

#### Registration

As with the clinic itself, the participants believed that the forums should be accessible to view content without registration particularly because “people don’t want to join forums unless they’ve seen what’s in them first.” [F1_P] However, participants agreed that students would have to have an account if they wanted to post: “You can’t interact with it unless you register.” [M8_FG] Participants believed that most students would be more likely to read posts than to post themselves and “you still want that information to be available because it’s useful for someone who doesn’t register.” [M8_FG]

## Discussion

### Principal Findings

Overall, one of the major themes that emerged from the current research related to the provision and treatment of personal information. The students in this study expressed the view that in most cases it should not be mandatory to provide personal information, particularly in the case of the most identifying or sensitive information, including mental health data. Previous research has demonstrated that students are very concerned about disclosing mental health information with university staff because they fear being identified as having a mental health problem [34]. However, the students in this study felt that it could be a barrier to use if it were not clear that the requested information was relevant to use of the service and being collected for a reason. Tailoring the site to the user was seen as an acceptable reason for requesting personal information. However, the students felt that the virtual clinic needed to explicitly demonstrate to the user the advantages of collecting such information. This is consistent with previous research that suggests users are more comfortable providing health information online when they can see clear benefits in doing so [27].

Some of the students in this study supported a model that reduced privacy in which users would be more identifiable to the university in exchange for the convenience of the integration of the virtual clinic with other online student services. Previous research has also found that easy access to health information can outweigh user concerns about privacy [29]. The students in this study felt that seamless integration, where they were provided with automatic access to an account linked across university services, could be highly desirable, particularly as it would avoid the stigma associated with actively signing up for a mental health service. Similarly, perceived stigma associated with disclosing mental health problems has been discussed previously as a barrier to face-to-face help-seeking from counseling centers by students [39].

A model whereby students were provided with access to services by the university would necessarily require that students were identifiable to the university on some level. Identification of users (via email at a minimum) would be necessary in the case of a student in crisis, as the university has duty of care for students [40]. In general, students were relatively comfortable with this model if they were convinced that the information could be kept secure and not provided to wider university staff not involved in running the clinic (ie, teaching staff) or to other people such as friends and family.

The privacy and security of personal information within the virtual clinic was considered an important issue for students. As noted by Vodicka et al [29] there are two separate risks involved: the privacy risks of having an online mental health account (ie, breaches of university security) and the risks of accessing these accounts online (ie, sharing university services passwords). The students maintained that this may not be a barrier to use if they were provided with a clear set of rules explaining the virtual clinic’s privacy and confidentiality processes. This is consistent with previous research examining electronic health portals which identified the need for clear explanations of consent, privacy ,and security processes to users [28,31]. Additionally, the need for privacy to be assured in order to feel comfortable using online services is consistent with other studies examining the development of health record resources [25,27].

The students’ views on many topics were not unified. However, one point they all agreed on was that personal choice was paramount in all aspects of the virtual clinic including anonymity in forums, release of personal information to the university, and sharing of information with professionals. Diversity in perspectives about privacy and releasing personal information has been found in previous qualitative research investigating health portals where some participants were more comfortable sharing information than others [27]. However, all participants in this study wanted assurance that no information would be released without their consent and even then that it would only be released to those who are central to their treatment on a need to know basis, similar to views expressed by community members in previous research [31].

### Limitations

There are two primary limitations that need to be considered for the current research. First, the aim of the two stages of this study was not specifically to collect data on privacy alone. Questions targeting privacy were asked within the context of a broader purpose of determining the content and functionality of the virtual mental health clinic, and the data were analysed ad hoc. It is possible that some aspects of privacy relating to the development of the virtual clinic were missed by using this method. Second, a relatively small self-selected group of students participated in the two stages of data collection. Therefore, the sample may not be representative other students at ANU or of the wider university student population.

### Conclusions

Privacy considerations are vital in the development of an online mental health virtual clinic for university students. Overall, the students believed that potential users of a university mental health clinic would be more comfortable providing data as long as they trusted the privacy and security of the website, particularly that their personal data will not be shared without their knowledge or consent.

This study highlights the potential difficulties of balancing user preferences with the requirements of the service provider. First, there are considerable challenges in implementing an online clinic that can integrate efficiently with other existing university services including student course accounts and connections to counseling centers or psychologists. Moreover, the expressed desire for personal choice in all aspects of the clinic (eg, preference to identify self on forums) also presents challenges and may not be able to be implemented due to safety or other concerns. Finally, the students occasionally held views that conflicted, for example, preferring to have an account set up by the university but desiring to be unidentifiable to the university. Overall, implications of this study suggest that the virtual clinic will need to be built flexibly to accommodate varying user preferences for levels of engagement and the provision of information and that privacy of user information will need to be balanced against other potential capabilities of the clinic.

## Appendix

Multimedia Appendix 1 Focus group and prototype session questions.
